# Odors Associated With Autobiographical Memory Induce Visual Imagination of Emotional Scenes as Well as Orbitofrontal-Fusiform Activation

**DOI:** 10.3389/fnins.2021.709050

**Published:** 2021-08-03

**Authors:** Yuri Masaoka, Haruko Sugiyama, Masaki Yoshida, Akira Yoshikawa, Motoyasu Honma, Nobuyoshi Koiwa, Shotaro Kamijo, Keiko Watanabe, Satomi Kubota, Natsuko Iizuka, Masahiro Ida, Kenjiro Ono, Masahiko Izumizaki

**Affiliations:** ^1^Department of Physiology, Showa University School of Medicine, Tokyo, Japan; ^2^Sensory Science Research, Kao Corporation, Tokyo, Japan; ^3^Department of Ophthalmology, Jikei University School of Medicine, Tokyo, Japan; ^4^Human Arts and Sciences Research Center, University of Human Arts and Sciences, Saitama, Japan; ^5^Department of Neurology, Showa University School of Medicine, Tokyo, Japan; ^6^National Hospital Organization Mito Medical Center, Ibaragiken, Japan

**Keywords:** autobiographical memory, functional magnetic resonance imaging, fusiform gyrus, odor, older adults, orbitofrontal cortex

## Abstract

Specific odors can induce memories of the past, especially those associated with autobiographical and episodic memory. Odors associated with autobiographical memories have been found to elicit stronger activation in the orbitofrontal cortex, hippocampus, and parahippocampus compared with odors not linked to personal memories. Here, we examined whether continuous odor stimuli associated with autobiographical memories could activate the above olfactory areas in older adults and speculated regarding whether this odor stimulation could have a protective effect against age-related cognitive decline. Specifically, we used functional magnetic resonance imaging to investigate the relationship between blood oxygen levels in olfactory regions and odor-induced subjective memory retrieval and emotions associated with autobiographical memory in older adults. In our group of healthy older adults, the tested odors induced autobiographical memories that were accompanied by increasing levels of retrieval and the feeling of being “brought back in time.” The strength of the subjective feelings, including vividness of the memory and degree of comfort, impacted activation of the left fusiform gyrus and left posterior orbitofrontal cortex. Further, our path model suggested that the strength of memory retrieval and of the emotions induced by odor-evoked autobiographical memories directly influenced neural changes in the left fusiform gyrus, and impacted left posterior orbitofrontal cortex activation through the left fusiform response.

## Introduction

Specific odors can induce autobiographical memories (AM-odor), which are accompanied by the visual experience of a spatial and emotional scene, feelings of “being brought back in time” to a moment in the past, heightened emotional arousal, and the sensation of comfort (Willander and Larsson, 2006). Compared with other sensory input such as auditory and visual cues, memory retrieval induced by a specific odor can induce powerful visual imagery (Herz, 2004). Brain imaging studies have indicated that AM-odors activate the hippocampus (HI) and amygdala (AMG) (Herz, 2004; Masaoka et al., 2012a), and in younger individuals, the posterior orbitofrontal cortex (POFC) as well (Watanabe et al., 2018). Because olfactory information is directly transmitted to the piriform cortex, AMG, and entorhinal cortex (ENT), that is, centers of emotion and memory processing, olfactory input to these areas is directly associated with individual emotional past experiences (Herz and Cupchik, 1992; Masaoka et al., 2012a). The information finally converges in the OFC, which plays a role in olfactory identification and conscious awareness of emotions. Indeed, the level of OFC activation elicited by an AM-odor might be associated with the strength of a particular memory, with parallel activation of the para-HI (Watanabe et al., 2018).

The left POFC plays a role in reward processing (Breiter et al., 2001) and motivation (Roberts et al., 2016). Consequently, activation of the POFC by an AM-odor might influence subsequent behavior or cognition, especially in older adults with declining cognitive function and/or olfactory ability. Olfactory ability is known to decline with age (Doty, 1989). In addition to age-related decline, olfactory impairment has been reported as a first sign of mild cognitive impairment (MCI) (Roberts et al., 2016) and is observed in patients with Alzheimer’s disease (Doty, 1989; Hawkes, 2003). Such olfactory impairment may be caused by pathological changes, including the accumulation of senile plaques and neurofibrillary tangles, which appear first in the HI and AMG (Mesholam et al., 1998). These areas are critical for functions of memory retrieval and reaction of emotion. Consequently, episodic memory function declines as people age (Park et al., 1996), and autobiographical memory has been found to be impaired in patients with Alzheimer’s disease (Fromholt and Larsen, 1991). In one study that used facial expression stimuli in an emotion recognition task, older people exhibited poorer emotion recognition performance than younger individuals (Grainger et al., 2017). Thus, emotion recognition may be an index of social function, and appears to decline with age. Given the observed age-related decreases in olfactory ability, memory, and social function, the stimulation of emotions and memory *via* olfaction might decrease the rate of cognitive change in older adults. Specifically, an odor associated with an autobiographical memory might stimulate emotion and memory retrieval, with corresponding activation of the AMG, HI, and OFC.

To test this hypothesis in the present study, we had two research objectives. First, we examined the brain areas associated with AM-odor in older adults. If specific odors are strongly linked to episodic and emotional memories, then exposure to these odors may enhance neural responses in specific areas of the brain. By setting these specific brain areas as regions of interest, we examined whether connectivity existed between these areas using psychological interaction analysis (PPI) (Friston et al., 1997). Second, we used path analysis to test how the identified brain areas interacted with subjective scale scores, thus enabling us to create a network model including subjective memory and emotions induced by odor.

## Materials and Methods

### Participants

The study participants comprised 30 older adults who were living independently and had self-reported memory loss and mild cognitive deficits. All subjects were recruited from the Meguro Human Resource Center, Tokyo Japan. An experienced neurologist assessed the subjects using the Japanese version of the Montreal Cognitive Assessment (MoCA-J) (Nasreddine et al., 2005). All participants had normal cognitive function (25.5 ± 2, indicated in Table 1; Fujiwara et al., 2010). No participants had a personal history of seizures or head injury, or a diagnosis of a neurological or psychiatric disorder.

**TABLE 1:** Demographic data of older adult participants.

Total (M/F), No.	24 (Female, 12/Male, 12)
Age, y	74.4 ± 0.7
Handedness	Right, 22, Left, 2
Years of education	13.9 ± 2.5
MoCA-J	25.5 ± 2
Olfactory detection	0.93 ± 0.74
Olfactory recognition	2.95 ± 1.5

*MoCA-J, Japanese version of the Montreal Cognitive Assessment.*

We excluded two subjects with cerebral infarction and one subject with a history of subarachnoid hemorrhage. Accordingly, 27 subjects underwent magnetic resonance imaging (MRI). Of these, two subjects were excluded because of technical issues and one resigned during the scanning processes because of discomfort. Thus, the final sample included 24 subjects. All 30 initial subjects took part in experimental sessions on two separate days. On the 1 day, we conducted interviews to determine medical history, olfactory ability, and cognitive ability, and conducted pre-testing to enable odor selection. On the 2 day, which took place 2 weeks after the 1 day, 27 subjects underwent MRI scanning.

All experiments were conducted in accordance with the Declaration of Helsinki https://www.wma.net/policies-post/wma-declaration-of-helsinki-ethical-principles-for-medical-research-involving-human-subjects/. The study was approved by the Ethical Committees of Showa University School of Medicine, and all participants provided written informed consent prior to participation.

### Olfactory Ability

We used the T&T olfaction test (Takasago International Corporation, Tokyo, Japan) to measure olfactory detection and recognition levels. This test is often used in the field of otolaryngology to test the ability to detect (able to smell) and recognize (identification or naming of odor) and is well correlated with the Pennsylvania Smell Identification Test (Kondo et al., 1998). The detailed methods are given in previous studies (Watanabe et al., 2018).

### Pre-testing for Odor Selection

During the pretest interviews on the first experimental day, we tested five odors to determine whether they would induce autobiographical memory in the participant group. The odors were tatami (Japanese straw mat), osmanthus flower, baby powder, citrus, and incense. These were the five odors that induced the strongest autobiographical memories in a previous study in which 30 volunteers were asked to describe memories and rate the level of emotions evoked by the stimuli (Masaoka et al., 2012b). In the present study, all of the participants were asked to describe the memories evoked by the five odors and to rate the strength of the associated emotions. The odors were presented in a randomized order. The participants were exposed to each odor, and then asked to complete the following questions:

(1) Does this odor elicit a specific memory associated with a person, place, or event?
(2) Which age was associated with the memory induced by the odor (younger than 10 years old, teenage years, 20s, 30s, 40s, 50s, 60s, and 70s)?
(3) Rate the degree of pleasantness felt when recalling the memory induced by the odor and the memory context (1 = very unpleasant; 5 = very pleasant).
(4) Rate the vividness of the memory context (1 = not at all strong; 5 = extremely strong).
(5) How strong was the feeling of being “brought back in time” to the occurrence of the event (1 = not at all strong; 5 = extremely strong)?
(6) How emotionally intense was the memory related to the odor (1 = not at all strong; 5 = extremely strong)?

Of the five odors, the one that induced the most powerful autobiographical memory for each participant was used as the AM-odor for that individual. As in a previous study, β-phenyl ethyl alcohol was used as a control odor (Masaoka et al., 2012a,b; Watanabe et al., 2018). The previous study reported that the odor of β-phenyl ethyl alcohol was similar to that of a rose odor, had the characteristics of a “normal odor,” and did not induce memory retrieval but had the same level of pleasantness as one of the AM-odors (Masaoka et al., 2012b; Watanabe et al., 2018).

### Imaging Data Acquisition

All imaging protocol were the same as in the previous study (Watanabe et al., 2018). MRI scanning (3 Tesla Magnetom Trio scanner, Siemens, Erlangen, Germany) was conducted at Ebara Hospital, Tokyo, Japan, and sessions took place between 6 and 8 pm on Mondays after a brief clinical examination. The scanner had a 32-channel head coil, and functional imaging was acquired with multiband accelerated gradient-echo echo planar imaging. To increase the temporal resolution, four slices were acquired simultaneously. The fMRI time series comprised 330 whole-brain volumes/session, each comprising 39 axial slices (matrix: 80 × 80; TR: 1 s; TE: 27 ms; FOV: 16–22 cm, thickness: 2.5 mm; flip angle: 90°). Anatomical MRI was also acquired using 3D-magnetization-prepared rapid-acquisition-by-gradient-echo T1-weighted sagittal sections.

Statistical analysis for the fMRI data was performed using statistical parametric mapping (SPM12) software (Wellcome Department of Cognitive Neurology, London, United Kingdom). The software was implemented in MATLAB (R2013B; Math Works Inc., Natrick, MA, United States) on a computer running Mac OS X Yosemite. We performed image pre-processing including motion correction, co-registration of functional and structural images, normalization, physiological noise correction (SPM8, Drifter Toolbox), and spatial smoothing with a 6-mm FWHM Gaussian filter.

### fMRI for Olfaction

The odor stimuli were delivered using the same custom-designed MRI-compatible system that has been previously described (Masaoka et al., 2014; Watanabe et al., 2018). The system delivers an odorant when the participant breathes in through a nose mask (ComfortGel Blue Nasal Mask 1070038, medium size, Philips Respironics, PA, United States). Each subject wore a nose mask fitted with a one-way valve unit that comprised a valve for inspiration and a valve for expiration. The inspiration valve was attached to three balloon valves, each of which was attached to an odor cassette. The odor cassettes contained the AM-odor, the control odor, and no odor, respectively. The inflation and deflation of the balloon controlled the outside scanner, and when the balloon valve was opened, the odor was delivered to the subject *via* their force of their own inspiration through the odor cassette. Respiratory rate and cardiac output were measured by a respiratory pressure sensor in the nose mask and a photoplethysmogram transducer (TSD200-MRI and PPG100C-MRI; Bio Pac, LA System, Japan), respectively. End-tidal CO_2_ was constant throughout the experiment (no-odor baseline, 4.5 ± 0.05, control odor, 4.6 ± 0.02, AM-odor, 4.62 ± 0.03, measured with an O_2_ and CO_2_ Analyzer, Acro System, Chiba, Japan). Respiration, cardiac output, and end-tidal CO_2_ data were stored in LabChart on PowerLab (ML846, AD Instruments, Aichi, Japan), and respiration and cardiac data were used to eliminate physiological noise in the preprocessing of the fMRI data.

As in our previously report, we conducted two fMRI sessions for each participant, one in which the AM-odor was interspersed with unscented air (no-odor baseline), and one in which the control odor (rose) was interspersed with unscented air (baseline). Each session comprised five unscented and five scented 30-s blocks. We designed our study to have 30-s presentations of scented and unscented air to minimize adaptation to the olfactory stimuli.

### Subjective Data Analysis

We compared scores on subjective scales measuring the degree to which the stimuli were perceived as pleasant and comfortable, as well as the arousal level, vividness of the memory, level of memory retrieval, and the degree to which the participant felt “brought back in time” between the AM-odor and control odor *via* non-parametric Mann-Whitney tests.

### fMRI Data Analysis

We generated SPM contrast images (first-level) by comparing the AM-odor and no-odor baseline condition, and the control-odor and no-odor baseline condition. At the group level for whole brain analysis, we performed one sample *t-*tests for the *AM-odor vs. no-odor baseline* and *control odor vs. no-odor baseline* comparisons. For this analysis, we applied voxel-wise correction with *P* < 0.05 and family-wise error (FWE) correction for the whole brain volume. We inferred the difference between the AM-odor and control odor conditions using a paired *t*-test within the whole brain mask and corrected multiple comparisons (FWE-corrected *p* < 0.05) based on minimum cluster-extent thresholds estimated using the AlphaSim (AFNI; National Institutes of Mental Health, Bethesda, MD, United States) permutation procedure. Cluster-extent probability distributions were estimated based on 1,000 permutations of simulated Gaussian noise with a spatial smoothness of 9.2, 9.1, and 8.9 within the analysis mask. The permutation procedure resulted in a minimum required cluster-threshold of 3,821 voxels.

For exploratory analysis, we conducted psychophysiological interaction (PPI) analysis with SPM 12 to investigate the functional connectivity between activated areas. As in our previous report, the posterior orbitofrontal cortex (POFC) was activated during the AM-odor trials, so the voxels corresponding to this region (MNI coordinates: *x* = −38, *y* = 22, *z* = −10, sphere 6 mm) were set as voxels of interest. Then, we performed PPI analysis to explore the functional connectivity between the left POFC and all other voxels. PPI analysis was first performed for individuals, and then the resulting contrast images were entered into a group analysis with a one sample *t*-test for testing *AM-odor* > *no-odor baseline* and *control-odor* > *no-odor baseline* comparisons across the whole brain. For all of the above analyses, a FWE-corrected *P* < 0.05 was applied.

Then, we investigated how the subjective scales of comfort, vividness, level of memory retrieval, and level of being “brought back in time” interacted with the activation level of the target areas. The activation areas that were statistically significant in the fMRI analysis and PPI analysis were the left POFC and left fusiform gyrus. We conducted path analysis to examine the causal connections between the BOLD signal of the left POFC, that of the left fusiform gyrus, and the subjective scale scores.

Raw mean BOLD signals were extracted from a 10-mm sphere at the MNI coordinates *x* = −38, *y* = 22, *z* = −10 (left POFC) and *x* = −20, *y* = −66, *z* = −14 (left fusiform gyrus) for AM-odor PPI. For this analysis, we used the MarsBaR ROI toolbox (SPM 8),^1^ and entered the data into SPSS Statistics (IBM SPSS Statistics, Version 23.0, IBM Corp, Armonk, NY, United States). Before the path analysis, a number of analyses were performed to refine the model. We determined the Pearson correlations between the BOLD signals from the left POFC, the left fusiform gyrus, and the scores on the subjective scales, and conducted a multiple regression with interaction analysis.

Path analysis is similar to multiple regression analysis except that path analyses can be used to estimate the relative importance and significance of hypothesized causal connections between sets of variables. The statistical significance of the path coefficients was determined using a function in a structural equation modeling (SEM) program (IBM SPSS Statistics Amos, Version 23.0, IBM Corp, Armonk, NY, United States). Because our purpose was to create a causal model with observed variables, we employed path analysis instead of SEM, which takes into account the effect of latent variables on observed variables. We assessed the path model variability according to the goodness of fit index (GFI) and the Bollen-Stine bootstrap method. A GFI close to 1 and a Bollen-Stine bootstrap *P* value >0.05 were adopted for the model.

## Results

### Demographic Data and Subjective Scales

**FIGURE 1:**
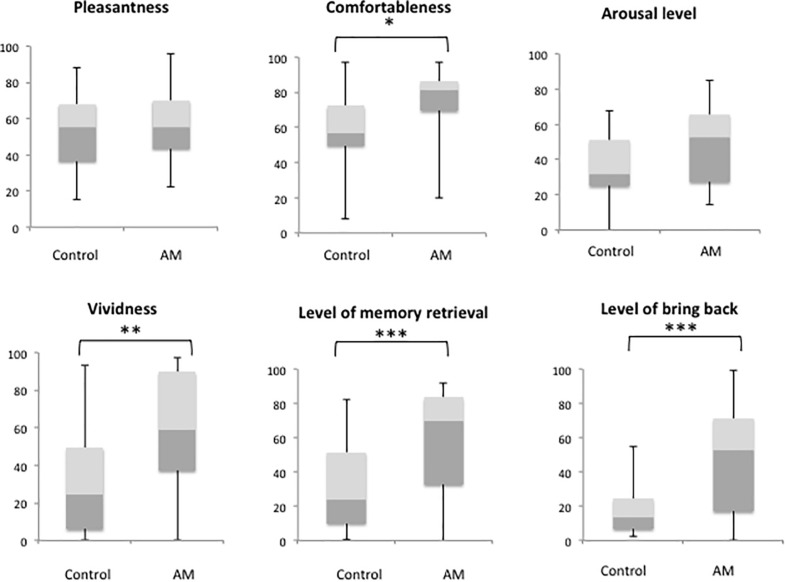
Comparison of subjective scale scores between trials with a control odor and those with an odor associated with an autobiographical memory (AM). *P < 0.05, **P < 0.01, ***P < 0.001.

Demographic data are given in Table 1. We confirmed that the olfactory detection and recognition scores were all in the normal range for older adults based on the data with that from age-matched patients with neurodegenerative disorders from our previous report (Masaoka et al., 2013). There was an equal amount of participants of each sex (men, 12, women, 12). A comparison between the AM-odor and control odor (Figure 1) indicated that the AM-odor significantly increased ratings of comfort (*z* = −2.1, *P* < 0.05), vividness (*z* = −2.52, *P* < 0.01), level of memory retrieval (*z* = −2.9, *P* < 0.001), and level of being “brought back to the past” (*z* = −0.32, *P* < 0.001) compared with the control odor. There were no differences in the degree of pleasantness (*z* = −0.56, *P* = 0.57) or arousal level (*z* = −1.84, *P* = 0.06) between the AM-odor and control odor trials.

### fMRI Results

**FIGURE 2:**
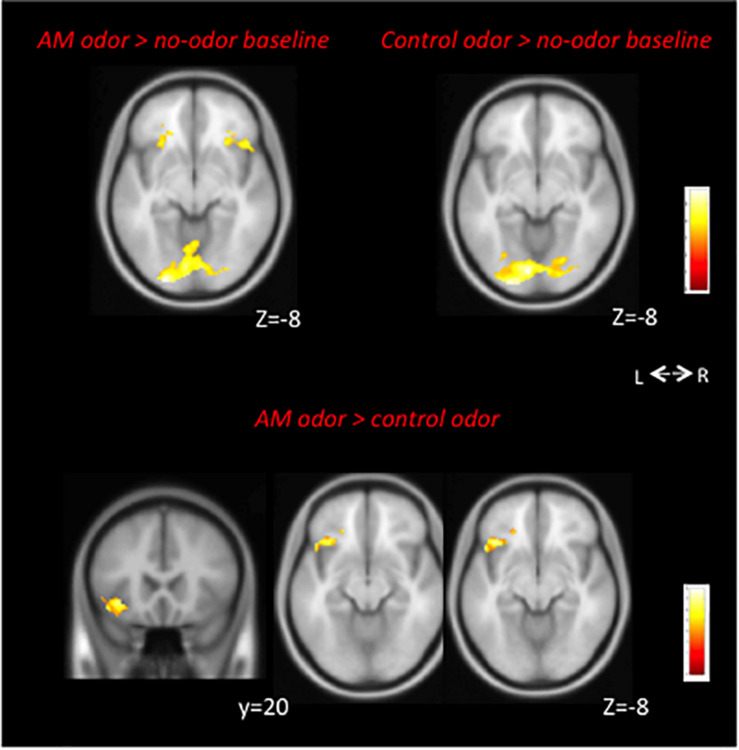
Top: Neural correlations in AM-odor (*AM-odor* > *no-odor baseline*, left) and control odor (*Control odor* > *no-odor baseline*, right) trials. Bilateral fusiform gyrus activation was common for both odors. Both results corresponded to FEW-corrected *P* < 0.05. We used *P* < 0.001 with 3,000 voxels for visual purposes. Bottom: The AM-odor significantly activated the left POFC compared with the control odor (bottom) at corrected significance thresholds corresponding to the whole-brain mask (FWE-corrected *p* < 0.05) (indicated peak cluster). L, left; R, right.

**FIGURE 3:**
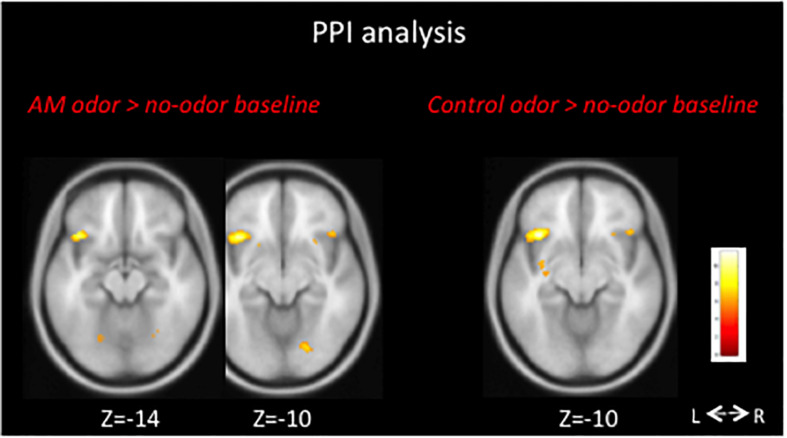
We performed PPI analysis to explore the functional connectivity between the left POFC and all other voxels. In AM-odor stimulation, activity in the left fusiform gyrus increased with activation of the left POFC (left). FWE corrected *P* < 0.05 was applied. L, left; R, right.

In the *AM-odor* > *no-odor baseline* comparison, the AM-odor trials had activation in the left POFC, right inferior frontal cortex, and bilateral fusiform gyrus (Figure 2 top left and Table 2A). In the *control odor* > *no-odor baseline* trials, we observed activation in the bilateral fusiform gyrus (Figure 2 top right and Table 2B). When comparing the AM-odor and control odor trials, the AM-odor significantly activated the left POFC compared with the control odor at corrected significance thresholds corresponding to the whole-brain mask (FWE-corrected *p* < 0.05) (indicated peak cluster in Figure 2 bottom and Table 2C, other cluster indicated in Supplementary Figure 1). The PPI analysis confirmed that activation of the left POFC area was associated with increased connectivity with the areas surrounding the left POFC, right POFC, and bilateral fusiform gyrus in the AM-odor trials, and that this was especially the case for the left fusiform gyrus (Figure 3). In the control odor trials, the left POFC appeared to be positively connected with the right POFC and the areas surrounding the bilateral POFC but not specifically the left fusiform gyrus. All cluster sizes, *z* scores, MNI coordinates, and brain regions associated with the PPI analysis are given in the Supplementary Table 1.

**TABLE 2:** Contrast fMRI data.

A: AM odor > no-odor baseline					

Peak voxel coordinate					
Cluster	Z scores	x	y	z	Regions
6	4.94	−24	−94	−8	L. fusiform
4	4.98	−18	−76	−18	L. fusiform
4	4.98	−28	22	−8	L. POFC
2	5.04	56	20	2	R. inferior frontal cortex
1	5.01	42	−62	−30	R. fusiform

**B: Control odor > no-odor baseline**					

**Peak voxel coordinate**					
**Cluster**	**Z scores**	**x**	**y**	**z**	**Regions**

14	5.04	−8	−82	−10	L. fusiform
10	4.96	26	−90	−2	R. fusiform
7	4.93	−18	−94	−6	L. fusiform

**C: AM odor > Control odor**					

**Peak voxel coordinate**					
**Cluster**	**Z scores**	**x**	**y**	**z**	**Regions**

4391	3.27	−36	21	−9	L. POFC
	3.2	−36	40	−4	L. inferior frontal
	3.2	−42	26	−8	L. inferior frontal

*POFC, posterior orbitofrontal cortex; L, left; R, right.*

### Path Analysis

According to above results, we analyzed the data from the left POFC and the bilateral fusiform gyrus using the following path analysis.

To investigate how the BOLD signals at the left POFC and the fusiform gyrus interacted with the subjective scales, we first tested our hypothesis. BOLD signals were extracted from the left POFC and the left fusiform gyrus in the AM-odor and control odor trials. Before the path analysis, we conducted Pearson correlation and multiple regression analysis to examine the relationship between each area in which we observed BOLD signal and the subjective scale scores. The statistical results of these correlation analyses are given in Supplementary Table 2 (AM-odor) and Table 3 (control-odor). In brief, in the AM-odor trials, we observed correlations between the BOLD signal in the left POFC and that in the left fusiform, and between the BOLD signal in the left fusiform gyrus and the level of memory retrieval and level of being “brought back in time.” The level of memory retrieval was correlated with the degree of comfort, vividness, and the level of being “brought back in time” (all *P* < 0.01). In the control odor trials, there was no correlation between activity in the left POFC, activity in the left fusiform gyrus (*P* > 0.05), and the subjective scale scores. The multiple regressions with interaction analysis revealed a significant interaction between the AM-odor and control odor trials in terms of the relationship between the left fusiform BOLD signal and the level of memory retrieval (β = 0.72, *t* = 1.96, *P* < 0.05), and between the left fusiform gyrus BOLD signal and the level of being “brought back in time” (β = 0.71, *t* = 1.95, *P* < 0.05) (Supplementary Figure 2). We observed no interaction for the relationships between activity in the left fusiform gyrus and vividness (β = 0.5, *t* = 1.6, *P* = 0.1) or degree of comfort (β = 0.9, *t* = 1.68, *P* = 0.09).

**TABLE 3:** Statistical results for the direct path in AM-odor trials.

Path	*r*	*P* value
Bring back — memory retrieval	0.83	*P* < 0.0001
Memory retrieval—L. fusiform	0.44	*P* = 0.02
L. fusiform—L. POFC	0.69	*P* < 0.0001
l. POFC—pleasant	0.33	*P* = 0.08
Comfortableness—bring back	0.61	*P* = 0.01
Comfortableness—vividness	0.8	*P* = 0.003
Bring back—vividness	0.8	*P* = 0.003

*Standardized regression weight, *p* value, correlations of covariances and *p* value.*

For path analysis, we first tested the full model including the path between the following variables: the left POFC, left fusiform gyrus, memory retrieval, degree of comfort, vividness, and level of feeling “brought back in time” for each the AM-odor and control odor trials.

**FIGURE 4:**
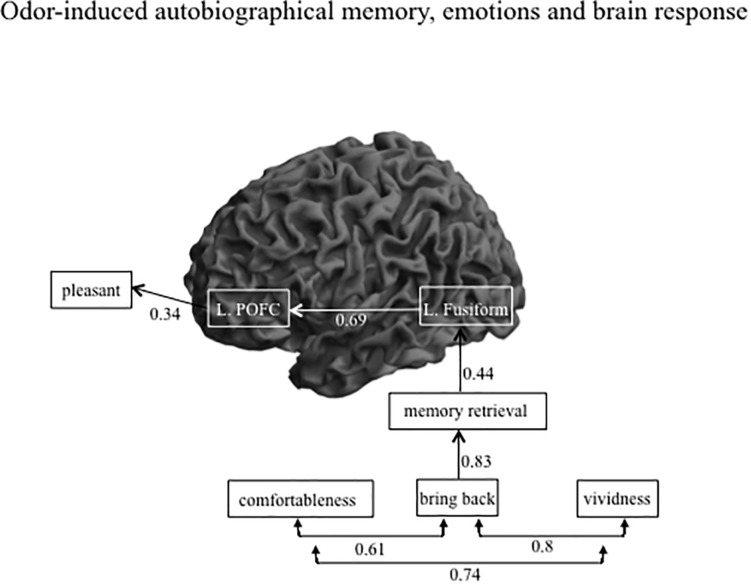
Path diagram showing the significant direct path for AM-odor trials. There were strong correlations among the subjective scales scores, and the level of feeling “brought back in time” had the strongest impact on memory retrieval. The strength of memory retrieval had a direct effect on activation in the left fusiform gyrus, and the level of activation of the left fusiform gyrus directly impacted that in the left POFC.

After eliminating non-significant paths, we determined the final path to have a GFI value of 0.9 with the Bollen-Stine bootstrap method, *p* = 0.24. Figure 4 shows the path diagram with a significant direct path for AM-odor. All statistical details are given in Table 3. The degree to which the participant felt they had been “brought back in time” had a significant path to memory retrieval (*r* = 0.8, *P* < 0.0001). Further, memory retrieval impacted the left fusiform gyrus (*r* = 0.44, *P* < 0.05), and the left fusiform gyrus had a direct path to the left POFC (*r* = 0.69, *P* < 0.0001). In terms of covariances, we found a correlation between the degree of comfort and the level of feeling “brought back in time” (*r* = 0.61, *P* < 0.05), between the degree of comfort and vividness (*r* = 0.74, *P* < 0.01), and between the level of feeling “brought back in time” and vividness (*r* = 0.8, *P* < 0.01). The other scales of pleasantness and arousal level were not statistically significant in the direct path results, and we found no significant direct path from the left POFC to pleasantness (*r* = 0.34, *P* = 0.08). However, given that the POFC was previously associated with pleasant emotion (Gottfried et al., 2002), we expected to find an indirect path from the variables.

**FIGURE 5:**
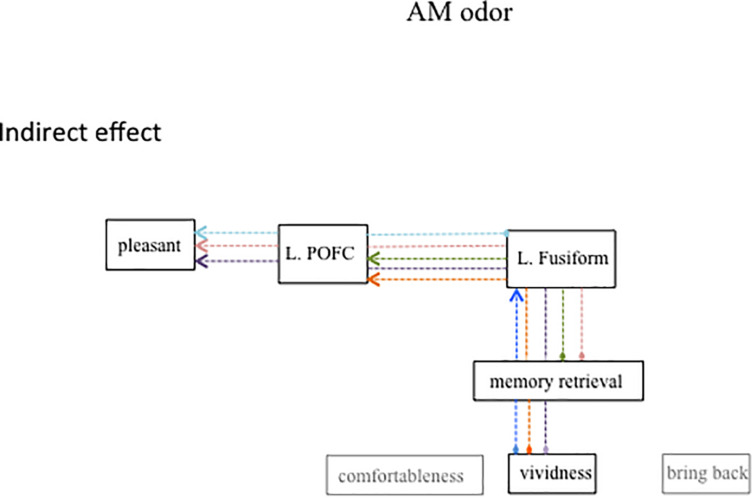
Path diagram showing significant indirect path for AM-odor trials. Each colored dot indicates the starting point of the indirect path and the same colored arrow indicates the final affect variable. Subjective scale scores indirectly affected the left POFC through the left fusiform gyrus. The strength of memory retrieval predicted the degree of pleasantness, which appeared to depend on the level of activation in the left fusiform gyrus and the left POFC.

Figure 5 shows the same diagram as in Figure 4 but includes the significant indirect path of AM-odor. The statistical results of the indirect path are given in Table 4. The level of vividness had an indirect effect on activity in the left fusiform gyrus through memory retrieval (blue dotted line). Further, the level of vividness was connected *via* an indirect path to the left POFC through memory retrieval and activity in the left fusiform gyrus (orange dotted line) and also had an indirect effect on pleasantness through memory retrieval, activity in the left fusiform gyrus, and activity in the left POFC (purple dotted line). The degree of memory retrieval had an indirect effect on activity in the left POFC through the left fusiform gyrus (green dotted line), and impacted pleasantness through the left fusiform and the left POFC (pink dotted line). Activity in the left fusiform gyrus also had an indirect effect on pleasantness through the left POFC (pale blue dotted line).

**TABLE 4:** Statistical results for the indirect path in AM-odor trials.

From	To	Lower bounds	Upper bounds	*P* value
Bring back	L. fusiform	0.11	0.59	*P* = 0.01
Bring back	L. POFC	0.03	0.5	*P* = 0.01
Bring back	Pleasant	0	0.25	*P* = 0.05
Memory retrieval	L. POFC	0.03	0.5	*P* = 0.02
Memory retrieval	Pleasant	−0.001	0.3	*P* = 0.05
L. fusiform	Pleasant	−0.01	0.54	*P* = 0.1

*Standardized regression weight, *p* value, correlations of covariances and *p* value.*

For the control odor, we found an direct path between the subjective scale scores but no significant direct or indirect path to the left fusiform gyrus (Supplementary Figure 3 and Supplementary Table 4).

## Discussion

In this study, we examined the relationship between activity in different brain regions and subjective emotional scale scores for acquired odors *via* functional imaging of the left POFC and the left fusiform gyrus during olfactory memory scanning sessions. We found that activation of the left POFC was associated with increased connectivity with the left fusiform gyrus, and path analyses indicated that these two regions were associated with memory retrieval and the feeling of being “brought back in time” during AM-odor trials. Notably, the subjective level of memory retrieval, vividness of the memory, and the associated degree of emotional comfort impacted the level of activation of the left fusiform gyrus and left POFC. Finally, the path model indicated that high subjective memory and emotion scale scores in the AM-odor trials were associated with activity in the left fusiform gyrus and the left POFC, as well as the degree to which the odor was rated as pleasant.

The role of the left POFC has been previous established in terms of reward processing (Breiter et al., 2001), response to attractive human faces (Aharon et al., 2001), and responses to pleasant odor stimuli (Gottfried et al., 2002). The left POFC is connected to the AMG, and the interaction between these two areas may involve emotional perception and awareness of enhanced emotions Murray and Izquierdo, 2007). In non-human primates, the caudal OFC is structurally connected to the AMG (Barbas, 2007), and the OFC receives input from temporal visual areas (Barbas, 1993). Indeed, in a human study, Frank et al. (2019) reported that an enhanced BOLD signal in the AMG was connected with activity in the OFC as well as the fusiform gyrus while participants viewed photos of an emotional scene. During this visual emotional perception, the neural response of the AMG was correlated with that of the fusiform gyrus, which is known to be a critical region for vision. These non-human primate and human studies support our path model, and the most interesting finding of the present study was that activation of the fusiform gyrus (as indicated by the BOLD response) was elicited not by visual but olfactory stimuli associated with autobiographical memory.

Odors are known to induce strong mental imagery (Herz and Cupchik, 1992). Further, an fMRI study reported that the visual perspective during retrieval of autobiographical memories activated the posterior part of the visual areas (St. Jacques et al., 2017). According to these studies, fusiform activation might also be associated with odor-induced visual imagery. Although our results indicated that the control odor also activated the fusiform gyrus, we expect that the connection between the POFC and fusiform gyrus would more strongly increased during memory retrieval and the feeling of being “brought back in time” during exposure to the AM-odor vs. the control odor. The odors in this study evoked imagery of autobiographical memories, including emotional episodic scenes and emotional special scenes, and the strength of these experiences was associated with the activation of the left fusiform gyrus and left POFC. Indeed, most of the subjects reported detailed descriptions of the evoked memories, for example, a memory of being a child playing with friends in a green field and smelling asmanthus flowers, or a memory of talking with their grandmother on a tatami mat, and so on. The fusiform gyrus is in the occipitotemporal gyrus, which is well known to be involved in the visual processing of human faces (Weiner and Grill-Spector, 2012), bodies (Peelen and Downing, 2005), and the perception of stimuli with high spatial frequency. Compared with the experience of recalling the memories without an odor, the scenes in the odor trials might have had an enhanced sense of realism and vividness, reflecting an enhanced fusiform-POFC connection.

One concern is that the anterior cingulate cortex (ACC) was not activated in our study. The ACC, POFC, and the anterior insula have previously been observed to exhibit activation at the same time as the primary olfactory cortex (Poellinger et al., 2001). García-Cabezas and Barbas (2014) suggested that the ACC might become activated at the same time as the primary olfactory cortex to mediate the process of rapid attention to olfactory stimuli, and to integrate this information with other sensory modalities *via* the POFC and anterior insula. In the present study, subjective feelings regarding the retrieved memories and the vividness of the memories were related to the strength of the activation of the fusiform gyrus and POFC. These results suggest that the subjects may have attended more to the retrieved memory than to the olfactory stimuli itself. Indeed, in a previous study, the HI and AMG were activated during retrieval of autobiographical memories *via* a specific odor (Herz, 2004). These data provide information regarding how the ACC integrates inhibition and excitation with activation of the parahippocampus, HI, POFC, and fusiform gyrus, although further research is needed.

Although the AMG, fusiform gyrus, and OFC had been proposed to have directional interconnectivity in emotional scene perception (Frank et al., 2019), the AMG was not included in the path analysis in our study.

Particularly, the AMG did not survive the GLM analysis with FWE correction. As in the previous study, the piriform cortex, AMG, and hippocampus, which are regarded as the primary olfactory areas, did not reach statistical significance. There are two possible reasons for this. First, in terms of primary olfactory fMRI activation, these regions may rapidly habituate to olfactory stimuli (Masaoka et al., 2014; Watanabe et al., 2018). Specifically, repeated presentation of the same odorant may lead to decreased neural responses in the PIR, ENT, and AMG, and in turn, a decreased BOLD response. Although the primary olfactory areas are known to habituate very quickly, downstream secondary areas of the OFC continue to respond to recurrent odor presentation (Poellinger et al., 2001; Sobel et al., 2001).

The other possibility is that the areas related to conscious awareness of emotion or memory, as well as visual spatial processing, had more powerful responses to the stimuli. Thus, the strong mental imagery might have strengthened the statistical power of activation in the frontal and occipital areas. Further studies that compare activation observed during odor-induced memory or emotion and that accompanied by visual stimuli are needed to more clearly understand the role of AMG activation.

As with previous reports, activation in the left OFC and left fusiform was more significant than that on the right side. Svoboda et al. (2006) indicated in a meta-analysis that autobiographical memory was associated with a network of left-lateralized structures including the medial and ventrolateral prefrontal regions. For instance, the left OFC was dominant during conscious assessment of the emotional quality of an odor (Royet et al., 2003). In terms of left fusiform activation, Spagna et al. (2021) reported that robust activity in the left fusiform gyrus was associated with visual mental imagery. Evidence from patients with brain damage indicates that visual mental imagery is impaired after extensive damage of the temporal lobe, especially that in the left hemisphere (Bartolomeo, 2002). Specifically, a case study of patients with selective damage to the right lingual gurus and left posterior medial fusiform gyrus revealed impaired visual mental imagery. The left fusiform activation observed in our study was located near the medial posterior regions, suggesting that this left fusiform area may be implicated in semantic and lingual processing associated with mental imagery, especially given that with the left OFC plays a role in conscious awareness of memory and emotion.

## Limitations

Several limitations should be noted. First, we analyzed a small number of subjects, and thus studies with a larger number of subjects, including those with MCI, are needed to clarify the path direction of brain activation in terms of subjective memory level and emotions. Second, although we used a subjective scale to measure odor-evoked autobiographical memory, the participants also reported that the odors elicited episodic and spatial memory. Further studies should include evaluations of autobiographical memories (Kopelman et al., 1989), as well as detailed descriptions of the retrieved memories. In addition, future studies should compare neural activities according to the characteristics of the evoked autobiographical memories. Third, we used a careful procedure to clean the odor stimuli from our experimental device, and used only two odors for each participant to reduce the risk that the odors would mix in the valve. In the future, we hope to develop an odor delivery system that enables the presentation of several AM-odors and control odors, as this would enable us to test the relationship between the neural response and subjective scales scores for each odor. Fourth, although we obtained an optimized path model, we could not determine the cause-and-effect relationships between points in the pathway. To investigate this relationship, a method with a higher time resolution such as electroencephalogram (Frank et al., 2019) might be helpful.

## Summary

Olfactory abilities are known to decline with age, and olfactory decline may predict further decreases in cognitive and emotional function (Roberts et al., 2016). Our data indicate that in healthy older adults, odors induced autobiographical memories along with activation of the left POFC. This result was consistent with our previous report, which involved young subjects. In older adults, an increased level of memory retrieval and feeling of being “brought back in time” accompanied activity in the left POFC and left fusiform gyrus. Subjective feelings such as the vividness of the memory and degree of comfort impacted the level of left fusiform gyrus and left POFC activation. Our path model suggested that the strength of the memory and emotions elicited by the odor-induced autobiographical memory, which was associated with neural changes in two brain regions, could indirectly impact subjective pleasantness. Activation of the POFC appears to be important for cognitive function and motivation (Watanabe et al., 2018), and retention of these functions is important for older adults. Glachet and El Haj (2019) suggested that odor exposure could be a useful tool for improving the quality of autobiographical memory retrieval and increasing the number of positive memories in patients with Alzheimer’s disease. Taken together, the existing data indicate that continuous stimulation with an odor associated with a strong memory may elicit a continuous neural response in the POFC, and that this neural response could play a role in protecting cognitive function. In the future, we plan to continue to explore how odors associated with autobiographical memory exposure might contribute to the clinical features of neurodegenerative disorders. However, more work is required to investigate whether odors that evoke positive memories and emotions can contribute to healthy aging in terms of all physiologically, cognitive and emotional function.

## Data Availability Statement

The original contributions presented in the study are included in the article/Supplementary Material, further inquiries can be directed to the corresponding author/s.

## Ethics Statement

The studies involving human participants were reviewed and approved by the Ethical Committees of Showa University School of Medicine. The patients/participants provided their written informed consent to participate in this study.

## Author Contributions

YM and HS designed the study and conducted experiments. YM analyzed the fMRI data and wrote the initial manuscript. HS analyzed subjective scale data. MY, AY, MH, NK, and KO recruited participants and assisted with scanning. MId assisted with manipulation of the fMRI device. KW, SKu, SKa, and NI performd pre-processing of the fMRI data. YM, HS, and MIz edited the final manuscript. All authors discussed the results.

## Conflict of Interest

The authors declare that this study was funded by the Kao Corporation, where HS was employed. HS was involved in the study design, execution, and analysis of subjective scale data. All data were analyzed by investigators who were blinded regarding the study groups.

## Publisher’s Note

All claims expressed in this article are solely those of the authors and do not necessarily represent those of their affiliated organizations, or those of the publisher, the editors and the reviewers. Any product that may be evaluated in this article, or claim that may be made by its manufacturer, is not guaranteed or endorsed by the publisher.

## Footnotes

1
http://marsbar.sourceforge.net

## References

[B1] Aharon I. Etcoff N. Ariely D. Chabris C. F. O’Connor E. Breiter H. C. (2001). Beautiful faces have variable reward value: fMRI and behavioral evidence. *Neuron* 32 537–551. 10.1016/s0896-6273(01)00491-3 11709163

[B2] Barbas H. (1993). Organization of cortical afferent input to orbitofrontal areas in the rhesus monkey. *Neuroscience* 56 841–864. 10.1016/0306-4522(93)90132-y 8284038

[B3] Barbas H. (2007). Flow of information for emotions through temporal and orbitofrontal pathways. *J. Anat.* 211 237–249. 10.1111/j.1469-7580.2007.00777.x 17635630 PMC2375774

[B4] Bartolomeo P. (2002). The relationship between visual perception and visual mental imagery: a reappraisal of the neuropsychological evidence. *Cortex* 38 357–378. 10.1016/s0010-9452(08)70665-8 12146661

[B5] Breiter H. C. Aharon I. Kahneman D. Dale A. Shizgal P. (2001). Functional imaging of neural responses to expectancy and experience of monetary gains and losses. *Neuron* 30 619–639. 10.1016/s0896-6273(01)00303-8 11395019

[B6] Doty R. L. (1989). Influence of age and age-related diseases on olfactory function. *Annu. N.Y. Acad. Sci.* 561 76–86. 10.1111/j.1749-6632.1989.tb20971.x 2525363

[B7] Frank D. W. Costa V. D. Averbeck B. B. Sabatinelli D. (2019). Directional interconnectivity of the human amygdala, fusiform gyrus, and orbitofrontal cortex in emotional scene perception. *J. Neurophysiol.* 122 1530–1537. 10.1152/jn.00780.2018 31166811 PMC6843098

[B8] Friston K. J. Buechel C. Fink G. R. Morris J. Rolls E. Dolan R. J. (1997). Psychophysiological and modulatory interactions in neuroimaging. *Neuroimage* 6 218–229. 10.1006/nimg.1997.0291 9344826

[B9] Fromholt P. Larsen S. F. (1991). Autobiographical memory in normal aging and primary degenerative dementia (dementia of Alzheimer type). *J. Gerontol.* 46 85–91. 10.1093/geronj/46.3.p85 2030279

[B10] Fujiwara Y. Suzuki H. Yasunaga M. Sugiyama M. Ijuin M. Sakuma N. (2010). Brief screening tool for mild cognitive impairment in older Japanese: validation of the Japanese version of the montreal cognitive assessment. *Geriatr. Gerontol. Int.* 10 225–232. 10.1111/j.1447-0594.2010.00585.x 20141536

[B11] García-Cabezas M. Á Barbas H. (2014). A direct anterior cingulate pathway to the primate primary olfactory cortex may control attention to olfaction. *Brain Struct. Funct.* 219 1735–1754. 10.1007/s00429-013-0598-3 23797208 PMC5028194

[B12] Glachet O. El Haj M. (2019). Emotional and phenomenological properties of odor-evoked autobiographical memories in Alzherimer’s disease. *Brain Sci.* 9:135. 10.3390/brainsci9060135 31185649 PMC6627121

[B13] Gottfried J. A. O’Doherty J. Dolan R. J. (2002). Appetitive and aversive olfactory learning in humans studied using event-related functional magnetic resonance imaging. *J. Neurosci.* 22 10829–10837. 10.1523/jneurosci.22-24-10829.2002 12486176 PMC6758414

[B14] Grainger A. S. Henry J. D. Phillips L. H. Vanman E. J. Allen R. (2017). Age deficits in facial affect recognition: the influence of dynamic cues. *J. Gerontol. B. Psychol. Sci. Soc. Sci.* 72 622–632. 26530079 10.1093/geronb/gbv100

[B15] Hawkes C. (2003). Olfaction in neurodegenerative disorder. *Mov. Disord.* 18 364–372. 10.1002/mds.10379 12671941

[B16] Herz R. S. (2004). A naturalistic analysis of autobiographical memories triggered by olfactory visual and auditory stimuli. *Chem. Senses* 29 217–224. 10.1093/chemse/bjh025 15047596

[B17] Herz R. S. Cupchik G. C. (1992). An experimental characterization of odor-evoked memories in humans. *Chem. Senses* 17 519–528. 10.3389/fnbeh.2014.00240 8564426

[B18] Kondo H. Matsuda T. Hashiba M. Baba S. (1998). A study of the relationship between the T&T olfactometer and the University of Pennsylvania smell identification test in a Japanese population. *Am. J. Rhinol.* 12 353–358. 9805536 10.2500/105065898780182390

[B19] Kopelman M. D. Wilson B. A. Baddeley A. D. (1989). The autobiographical memory interview: a new assessment of autobiographical and personal semantic memory in amnesic patients. *Clin. Exp. Neuropsychol.* 11 724–744. 10.1080/01688638908400928 2808661

[B20] Masaoka Y. Harding I. H. Koiwa N. Yoshida M. Harrison B. J. Lorenzetti V. (2014). **T**he neural cascade of olfactory processing: a combined fMRI-EEG study. *Respir. Physiol. Neurobiol.* 204 71–77. 10.1016/j.resp.2014.06.008 24973471

[B21] Masaoka Y. Pantelis C. Phillips A. Kawamura M. Mimura M. Minegishi G. (2013). Markers of brain illness may be hidden in your olfactory ability: a Japanese perspective. *Neurosci. Lett.* 549 182–185. 10.1016/j.neulet.2013.05.077 23769725

[B22] Masaoka Y. Sugiyama H. Katayama A. Kashiwagi M. Homma I. (2012a). Remembering the past with slow breathing associated with activity in the parahippocampus and amygdala. *Neurosci. Lett.* 521 98–103. 10.1016/j.neulet.2012.05.047 22668857

[B23] Masaoka Y. Sugiyama H. Katayama A. Kashiwagi M. Homma I. (2012b). Slow breathing and emotions associated with odor-induced autobiographical memories. *Chem. Senses* 37 379–388. 10.1093/chemse/bjr120 22230171

[B24] Mesholam R. I. Moberg P. J. Mahr R. N. Doty R. L. (1998). Olfaction in neurodegenerative disease: a meta-analysis of olfactory functioning in Alzheimer’s and Parkinson’s diseases. *Arch. Neurol.* 55 84–90. 10.1001/archneur.55.1.84 9443714

[B25] Murray E. A. Izquierdo A. (2007). Orbitofrontal cortex and amygdala contributions to affect and action in primates. *Ann. N.Y. Acad. Sci.* 1121 273–296. 10.1196/annals.1401.021 17846154

[B26] Nasreddine Z. S. Phillip N. A. Bédirian V. Charbonneau S. Whitehead V. Collin I. (2005). The Montreal cognitive assessment, MoCA: a brief screening tool for mild cognitive impairment. *J. Am. Geriatr.* 53 695–699. 10.1111/j.1532-5415.2005.53221.x 15817019

[B27] Park D. C. Smith A. D. Lautenschlager G. Earles J. L. Frieske D. Zwahr M. (1996). Mediators of long-term memory performance across the life span. *Psychol. Aging* 11 621–637. 10.1037/0882-7974.11.4.621 9000294

[B28] Peelen M. V. Downing P. E. (2005). Selectivity for the human body in the fusiform gyrus. *J. Neurophysiol.* 93 603–608. 10.1152/jn.00513.2004 15295012

[B29] Poellinger A. Thomas R. Lio P. Lee A. Makris N. Rosen B. R. (2001). Activation and habituation in olfaction -An fMRI study. *Neuroimage* 13 547–560. 10.1006/nimg.2000.0713 11305885

[B30] Roberts R. O. Christianson T. J. H. Kremers W. K. Mielke M. M. Machulda M. M. Vassilaki M. (2016). Association between olfactory dysfunction and amnestic mild cognitive impairment and Alzheimer disease dementia. *JAMA Neurol.* 73 93–101. 10.1001/jamaneurol.2015.2952 26569387 PMC4710557

[B31] Royet J. P. Plailly J. Delon-Martin C. Kareken D. A. Segebarth C. (2003). fMRI of emotional responses to odors: influence o hedonic valence and judgment, handedness, and gender. *Neuroimage* 20 713–728. 10.1016/s1053-8119(03)00388-4 14568446

[B32] Sobel N. Prabhakaran V. Zhao Z. Desmond J. E. Glover G. H. Sullivan E. V. (2001). Time course of odorant-induced activation in the human primary olfactory cortex. *J. Neurophysiol.* 83 537–551. 10.1152/jn.2000.83.1.537 10634894

[B33] Spagna A. Hajhajate D. Liu J. Bartolomeo P. (2021). Visual mental imagery engages the left fusiform gyrus, but not the early visual cortex: a meta-analysis of neuroimaging evidence. *Neurosci. Biobehav. Rev.* 122 201–217. 10.1016/j.neubiorev.2020.12.029 33422567

[B34] St. Jacques P. L. Szpunar K. K. Schacter D. L. (2017). Shifting visual perspective during retrieval shapes autobiographical memories. *Neuroimage* 148 103–114. 10.1016/j.neuroimage.2016.12.028 27989780 PMC5344759

[B35] Svoboda E. McKinnon M. C. Levine B. (2006). The functional neuroanatomy of autobiographical memory: a meta-analysis. *Neuropsychologia* 44 2189–2208. 10.1016/j.neuropsychologia.2006.05.023 16806314 PMC1995661

[B36] Watanabe K. Masaoka Y. Kawamura M. Yoshida M. Koiwa N. Yoshikawa A. (2018). Left posterior orbitofrontal cortex is associated with odor-induced autobiographical memory: an fMRI study. *Front. Psychol.* 9:687. 10.3389/fpsyg.2018.00687 29867658 PMC5958215

[B37] Weiner K. S. Grill-Spector K. (2012). The improbable simplicity of the fusiform face area. *Trends Cogn. Sci.* 16 251–254. 10.1016/j.tics.2012.03.003 22481071

[B38] Willander J. Larsson M. (2006). Smell your way back to childhood: autobiographical odor memory. *Psychon. Bull. Rev.* 13 240–244. 10.3758/bf03193837 16892988

