# Supplementary material for: Odors Associated With Autobiographical Memory Induce Visual Imagination of Emotional Scenes as Well as Orbitofrontal-Fusiform Activation
Source: Front Neurosci. 2021 Aug 3;15:709050. doi: 10.3389/fnins.2021.709050 (PMC8369471; doi:10.3389/fnins.2021.709050)
Supplement: Supplementary file 1 [file Data_Sheet_1.docx]

Supplementary Material

**Odors associated with autobiographical memory induce visual imagination of emotional scenes as well as orbitofrontal-fusiform activation.**

Supplemental Figure 1

AM-odor significantly activated the left POFC compared with the control odor (peak cluster indicated in the main text) at corrected significance thresholds corresponding to the whole-brain mask (FWE-corrected p < 0.05). Other responded areas indicated in Supplemental Figure 1. L, left, R, right.

Supplemental Table 1 PPIs of the POFC and whole brain regions

*AM odor > no-odor baseline*

*Control odor > no-odor baseline*

Abbreviations: PPI, Psychophysiologic interaction analysis; POFC, posterior orbitofrontal cortex; L, left; R, right.

Supplemental Table 2 Results of Pearson correlation between targeted brain areas and subjective scales of AM-odor stimulation.

*P<0.05, **P<0.001

Supplemental Table 3 Results of Pearson correlation between targeted brain areas and subjective scales of control odor stimulation.

*P<0.05, **P<0.001

Supplemental Figure 2

The multiple regressions with interaction analysis revealed a significant interaction between the AM-odor and control odor trials in terms of the relationship between the left fusiform bold signal and the level of memory retrieval (β = 0.72, t = 1.96, P < 0.05), and between the left fusiform gyrus bold signal and the level of being ‘brought back in time’ (β = 0.71, t = 1.95, P < 0.05).

Supplemental Figure 3

Path analysis of control odor. For the control odor, we found an direct path between the subjective scale scores but no significant direct or indirect path to the left fusiform gyrus.

Supplemental Table 4　　Statistical results for the direct path in control odor trials.

Standardized regression weight, p value, correlations of covariances and p value.
